# Moderating effect of sleep quality in the relationship between coping and distress among medical students

**DOI:** 10.3389/fpsyt.2024.1259842

**Published:** 2024-10-02

**Authors:** Shahida Perveen, Najma Iqbal Malik, Muhammad Ebad ur Rehman, Muhammad Younas Khan, Syeda Tayyaba Rehan, Muhammad Sohaib Asghar, Amir H. Pakpour, Mark Griffiths, Irfan Ullah, Mohsin Atta

**Affiliations:** ^1^ Department of Psychology, University of Sargodha, Sargodha, Pakistan; ^2^ Deparment of Medicine, Rawalpindi Medical University, Rawalpindi, Pakistan; ^3^ Deparment of Medicine, Institute of Kidney Diseases, Peshawar, Pakistan; ^4^ Deparment of Medicine, Dow University of Health Sciences, Karachi, Pakistan; ^5^ Deparment of Medicine, Mayo Clinic, Rochester, MN, United States; ^6^ Social Determinants of Health Research Center, Research Institute for Prevention of Non-Communicable Diseases, Qazvin University of Medical Sciences, Qazvin, Iran; ^7^ Department of Nursing, School of Health and Welfare, Jönköping University, Jönköping, Sweden; ^8^ Psychology Department, Nottingham Trent University, Nottingham, United Kingdom; ^9^ Kabir Medical College, Gandhara University, Peshawar, Pakistan

**Keywords:** insomnia, sleep quality, psychological distress, coping, medical students

## Abstract

**Introduction:**

The present study examined the moderating effects of sleep quality in the relationship between coping and distress among medical college students. Present study was conducted to ensure the mental health of medical students and to dig out the reasons behind their disturbed health which can directly impact their performance at work.

**Methods:**

The study utilized a cross-sectional survey and was distributed to students at various medical institutions in the Punjab province of Pakistan from October 2019 to June 2020. The sample comprised 369 participants (120 males; 32.5%). The survey included the Pittsburgh Sleep Quality Index (PSQI), Kessler Scale of Psychological Distress (K10), Brief Cope Scale, and Wong and Law Emotional Intelligence Scale.

**Results:**

The results showed there was a significant relationship between coping and distress. More specifically, adaptive coping and distress were negatively associated (*r*=-.24), and maladaptive coping and distress were positively associated (*r*=.46). Moreover, the present study found that poor sleep quality was a significant positive predictor of distress. Moderation analysis showed that sleep quality was a significant moderator in the relationship between adaptive coping and distress (ΔR^2^=.011, β=-.36, *p*<.01) as well as between maladaptive coping and distress (ΔR^2^=.021, β=-.17, *p*<.01).

**Conclusion:**

The study’s findings clearly showed that sleep quality is a significant moderator in the relationship between coping (both adaptive and maladaptive) and distress among medical students.

## Introduction

Sleep is important for human development, and has been defined as a repetitive, reversible, and active condition related to disengagement of an individual’s perception with their environment and being unresponsive toward their surroundings (1, 2). According to National Sleep Foundation guidelines, it has been advised that average number of hours sleep needed for adolescents (aged 14-17 years) is 8-10 hours; young adults (18-25 years) is 7-9 hours (3); and college and university students is 7 hours and 45 minutes (4). Empirical evidence suggests disturbed sleep in approximately 60% of college and university students as assessed using the Pittsburgh Sleep Quality Index (PSQI), and that they delay bedtime and wakeup time at weekends (5; 6). Moreover, it has also been reported that students tend to take prescription drugs, recreational psychoactive drugs, and over-the-counter drugs to alter their wakefulness and sleep (7).

Students experiencing poor sleep quality face many physiological and psychological problems compared with those students having good sleep quality (3). In general, sleep quality is directly linked with several psychological and psychiatric disorders (8), in particularly with major depression through the melatonergic system (9). It has also been reported that perceived academic-related stress and emotional issues negatively influences student sleeping patterns (10). Previous research has also reported that stress and tension accounted for 24% of the change in PSQI score (3). In sum, university students are likely to encounter sleep problems and this may due to their transition to university life. More specifically, transition to university comes with various challenges (e.g., more difficult study, less supervision by parents, new social opportunities, etc.) which can cause sleep deprivation and disturbed schedules of sleep, especially among students who study professional majors such as medicine (11). The Pittsburgh Sleep Quality Index (PSQI) is considered as better instrument utilized for measuring the quality as well as patterns of sleep among adults. It can differentiate good quality from poor sleep quality through measurement of various aspects of sleep disturbance during previous month. Comparatively, The Epworth Sleepiness Scale (ESS) assesses individual’s level of sleepiness during daytime generally (12).

Among students who study professional majors, medical students are a cohort who report high perceived stress (13). Some scholars outlined “A Call to Action” to tackle the high perceived stress experienced by medical students (14). Study-related sources of stress reported by medical students include having tight time schedules, having high workloads, engaging in the dissection of corpses, seeing suffering patients, seeing dying patients, having contact with the severely ill, and having financial issues, alongside communication difficulties, language barriers, and cultural differences specifically in case of international students (14, 15). Over 40 years of research has shown that medical college students report a high degree of perceived stress and use different ways to cope with this stress on an individual level, alongside interventions such as stress reduction training (16), peer-group support programs (17), student-oriented curricula (18), and courses related to wellness (19). In sum, coping is important among medical students to help counteract their high levels of perceived stress.

Coping is an individual’s tendency to deal adequately with something troublesome (20). Coping is the cognitive and behavioral effort made by individuals to deal with stressful circumstances. Literature supports the fact that while coping with stressors, optimism and self-efficacy are personal resources that work as buffers for perceived stress (14). Other studies have noted that medical students who experience high levels of stress during the first year use active coping styles at medical school (21). Another study (22) proposed an interactive model for perceived stress among students on basis of literature postulating that personal factors (e.g., coping strategies or personality characteristics), and factors relevant to medical training (e.g., ethical concerns, workload, curriculum) were found to be antecedents of psychological distress. Possible personal experiences (e.g., substance abuse, relationship termination, suicidal ideation), as well as professional experiences (e.g., unsatisfactory academic performance, decrease in empathy, and other faults in medical training/professional attitude) and their interaction, were also found to be as consequences and determinants of psychological distress (23).

There is much worldwide empirical research that sleep quality is a significant predictor and moderator of maladaptive coping, such as alcohol use (24). The predictive relationship between sleep quality and maladaptive coping, including excessive internet use and drinking alcohol has been much reported (25, 26). A recent study (27) examining the relationship between poor sleep quality and stress utilizing multivariate analysis showed psychological distress to be a significant predictor of poor sleep quality among youth (28). Given these findings, the present study posited a model where sleep quality was proposed to be a moderator between coping and distress. To the best of the authors’ knowledge, these proposed relationships have never been studied in previous research and highlights the importance of the present study. Based on the aforementioned previous literature, the following hypotheses were formulated:

- Sleep quality would be negatively associated with distress (H_1_).
- Adaptive coping would be positively associated with sleep quality and maladaptive coping would be negatively associated with sleep quality (H_2_).
- Adaptive coping would be negatively associated psychological distress and maladaptive coping would be positively associated with psychological distress (H_3_).
- Sleep quality would be a significant moderator in the relationship between coping (adaptive and maladaptive) and psychological distress (H_4_).

## Method

### Study design and sample

A total of 369 medical students (120 males and 249 females) aged from 19 to 30 years, were selected utilizing convenience sampling from various medical colleges in the Punjab province of Pakistan. The study utilized a cross-sectional survey to collect the data.

### Place and duration of study

Only those students who majored in medicine at college in their first to fifth year were eligible to participate. Those students doing postgraduate training or a house job were excluded from the sample. A sample of students from various medical education colleges in Gujarat, Sargodha, and Lahore cities were selected. Data collection took place from October 2019 to June 2020.

### Procedure

The data were collected by a research team at the department of psychology, University of Sargodha, Pakistan. Proper procedures for permission from the institutional research review board and ethics committee of the department were followed. Moreover, the Postgraduate Research Board and Ethical Review Committee at the University of Sargodha approved the study (Ref/SU/PSY/789-5). Data were collected from Nawaz Shareef Medical College (in Gujarat), Akhter Saeed Medical College (in Lahore), and Sargodha Medical College (in Sargodha). The research team contacted the students directly. Official permission to collect data from all the medical colleges was granted. All student participants were told the purpose of the present study. The confidentiality and anonymity of data were ensured, and informed consent was obtained from participants before data collection. Detailed instructions were provided about how to complete the survey. Booklets were handed over to participants and they were requested to read the given instructions carefully. On average, students took 25 to 30 minutes to complete the survey.

### Measures

*The Pittsburgh Sleep Quality Index* (PSQI; 29; Urdu version: 30). The 19-item PSQI (comprising seven components: subjective sleep quality, sleep latency, sleep duration, sleep efficiency, sleep disturbance, use of sleep medications and daytime dysfunction) was used to assess sleep disturbances and sleep quality over a time interval of one month. Items (e.g., “During the past month, how often have you had trouble sleeping because you cannot get to sleep within 30 minutes?”) are rated from 0 (*not during the last month*) to 3 (*three or more times a week*). The total global sleep quality score is calculated by summing up the seven component scores and a global score of 5 and/or above is classified as “poor” sleep. The Cronbach’s alpha value in the present study was.80.

*Kessler Scale of Psychological Distress* (K10; 31; Urdu version: 32). The 10-item K10 was used to assess psychological distress. Items (e.g., “During the last 30 days, about how often did you feel tired out for no good reason?”) are rated from 1 (*none of the time*) to 5 (*all of the time*). The total score was calculated by summing up the scores on each item where the minimum value (i.e., 10) means no distress at all and the maximum value (i.e., 50) means distress is severe. The Cronbach’s alpha value in the present study was 0.91.

*Brief COPE* (33; Urdu version: 34). The 28-item Brief COPE was used to assess coping. The scale has 14 subscales with two items per subscale (*problem-focused coping* [comprising planning, use of instrumental support], *emotion-focused coping* [comprising religion, use of emotional support, positive reframing], *coping mechanisms, probably adaptive* [comprising acceptance, humor], *other coping mechanisms, probably maladaptive* [comprising venting, behavioral disengagement, mental disengagement, substance use, self-blame, denial)]. Items (e.g., *“I’ve been using alcohol or other drugs to make myself feel better”*) are rated from 1 (“I haven’t been doing it at all”) to 4 (“I have been doing it a lot”). In the present study, Cronbach’s alpha value for total scale was α=.86, while for its subscales the alpha values for both subscales i.e., maladaptive coping and adaptive coping was found as α=.80.

### Statistical analysis

Collected data were initially screened to exclude those surveys which were incomplete or randomly filled in. Data were then recorded in form of datasheets in SPSS-20. The responses from 369 medical students were analyzed to test the hypotheses. For statistical analysis, Pearson’s correlation tests and multiple linear regression were utilized to assess the relationships (see **Table 1**). To check the moderating effect of sleep quality on the relationship of coping (adaptive and maladaptive coping and distress, a hierarchal regression analysis was carried out. In model 1, we built a univariate linear regression model for crude estimates without incorporating moderation of sleep quality in the relationship between adaptive coping strategies and psychological distress. Model 2 utilizes the effect of sleep quality on multiple linear regression analysis to explain the variance of predictors in distress.

**Table 1:** Moderating role of sleep quality in the relationship of maladaptive coping and distress; adaptive coping and distress (N=369).

Variables	Model 1			Model 2		
	B	β	SE	B	β	SE
Constant	25.00***		.44	25.26***		.44
**Maladaptive coping**	4.28***	.45***	.45	4.27***	.45***	.45
Sleep quality	.46	.05	.45	1.22*	.13*	.51
MC*SQ				1.24***	.17***	.39
R^2^	.21			.23		
ΔR^2^				.02		
Constant	25.00***		.48	25.12***		.48
**Adaptive coping**	2.16***	.23***	.48	1.95***	.21***	.49
Sleep quality	1.12*	.12*	.48	1.66**	.17**	.55
AC*SQ				-1.13*	-.12*	.55
R^2^	.07			.08		
ΔR^2^				.01		

MC, maladaptive coping; AC, adaptive coping; SQ, sleep quality.

*p<0.05. **p<0.01. ***p<0.001.

## Results

Correlations between the main study variables are presented in **Supplementary Table 1**. The results suggest that poor sleep quality was significantly positively associated with distress and overall coping. However, poor sleep quality had a significant negative association with adaptive coping whereas it was significantly positively associated with maladaptive coping styles. Furthermore, distress was significantly negatively associated with the adaptive coping style and significantly positively associated with maladaptive coping style.

**Table 1** shows that sleep quality was a significant moderator in the relationship between maladaptive and adaptive coping with distress. In Model 1, the slope and R^2^ value of.21 showed that the predictors explained 21% variance in distress (F[2,366]=49.51, *p*<.001). The findings showed that maladaptive coping positively predicted distress (β=.45, *p*<.001), whereas sleep quality was non-significant at this stage (β=.05, *p*=.31).

In Model 2, the slope and R^2^ value of.23 showed that the predictors explained 23% variance in distress (F[3,365]=37.25, *p*<.001). The findings showed that maladaptive coping (β=.45, *p*<.001) and sleep quality (β=.13, *p*<.05) positively predicted distress at this stage. MC*SQ positively predicted distress (β=.17, *p*<.001). The ΔR^2^ value of.02 showed a 2% change in variance between Model 1 and Model 2 (ΔF[1,365]=10.24, *p*<.001). Analysis showed that sleep quality positively predicted the relationship between maladaptive coping and distress.

The second part of **Table 1** presents the moderation of sleep quality in the relationship between adaptive coping strategies and psychological distress. In Model 1, the slope and R^2^ value of.07 showed that the predictors explained 7% variance in distress (F[2,366]=13.93, *p*<.001). The findings showed that adaptive coping (β=.23, *p*<.001) and sleep quality (β=.12, *p*<.05) negatively predicted distress.

In Model 2, the slope and R^2^ value of.08 showed that the predictors explained 8% variance in distress (F[3,365)=10.79, *p*<.001). The findings showed that the adaptive coping (β=.21, *p*<.001) and sleep quality (β=.17, *p*<.01) positively predicted distress. AC*SQ negatively predicted distress (β=-.12, *p*<.05). The ΔR^2^ value of.01 showed a 1% change in variance between Model 1 and Model 2 (ΔF[1,365]=4.28, *p*<.001). Analysis showed that sleep quality positively predicted the relationship between adaptive coping and distress.

## Discussion

The present study explored the relationship between coping (both adaptive and maladaptive) and psychological distress. Correlational analysis supported the study hypothesis that coping (r= -.39**) (both adaptive and maladaptive) was associated with psychological distress. More specifically, maladaptive coping was significantly positively associated with psychological distress (r= .46**), whereas adaptive coping was significantly negatively associated with psychological distress (r= -.24**). These findings support a previous study (35) which found that the type of coping strategies used in changing situations contributed significantly to the prediction of perceived stress management among employees. Furthermore, the present study found that adaptive coping strategies predicted effective stress management but using maladaptive ways of coping decreased the effectiveness of stress management.

Regarding the relationship between sleep quality and psychological distress, the second hypothesis (i.e., sleep quality would be negatively associated with psychological distress) was supported by the present study’s findings (r= -.26**). These findings are also in line with a previous study (36) which concluded that poor sleep quality was associated with worsening psychological distress. Another study (25) also concluded that social media addiction and internet gaming disorder were both positively related to psychological distress as well as poor sleep quality.

The present study also examined the relationship between coping and sleep quality and the hypothesis that overall coping (r= .11**) would be significantly associated with sleep quality was supported. Correlational analysis indicated that the adaptive coping style (r= -.06*) was significantly negatively associated with poor sleep quality whereas the maladaptive coping style (r= .14**) was significantly positively associated with poor sleep quality. This relationship was discussed in a previous study (26) which evaluated the role of coping strategies in the relationship between sleep and stress. That study found that a high level of emotion-focused (i.e., maladaptive) coping predicted a loss of sleep time during high-stress periods. This implies that strategies used for coping are a major element to consider during sleep and stress research in the future.

**Figure 1:**
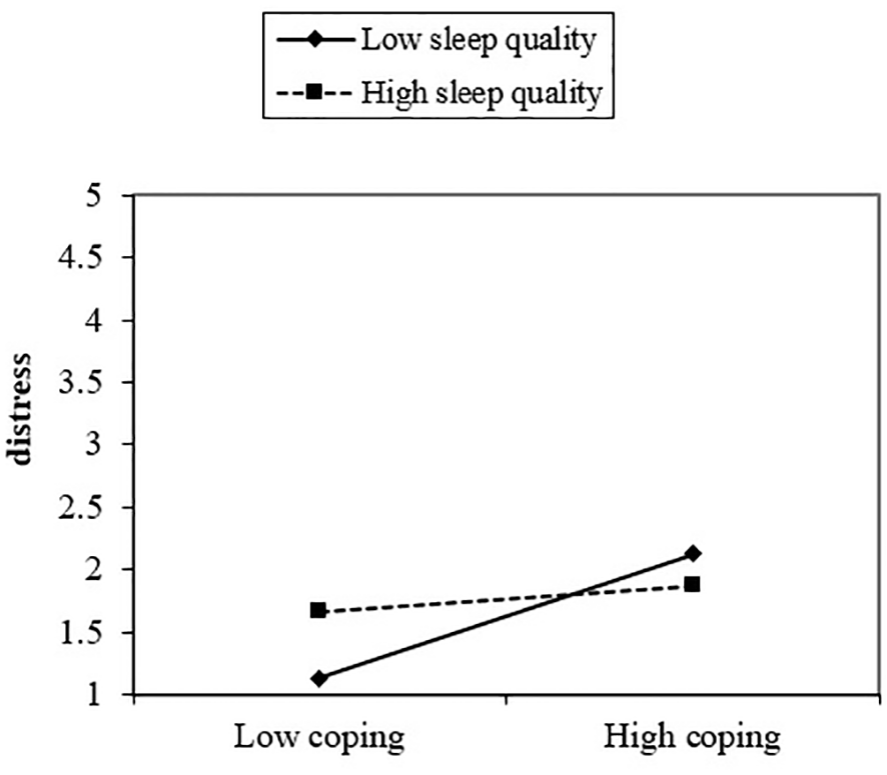
Sleep quality as a moderator in the relationship between coping and distress.

**Figure 2:**
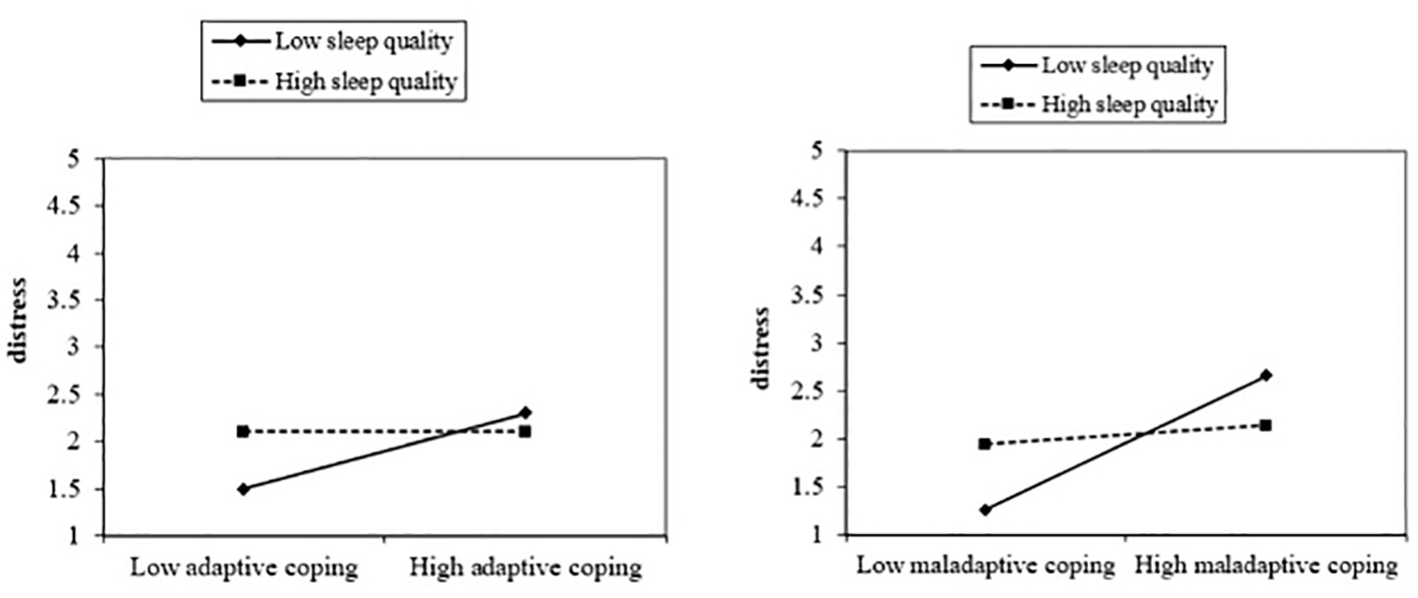
The moderating role of sleep quality in the relationship of adaptive coping and maladaptive coping with distress.

Finally, the last hypothesis that sleep quality would be a significant moderator in the relationship between coping (adaptive and maladaptive) and distress was also supported by the present study’s findings. Results from moderation analysis showed sleep quality to be a significant moderating variable between coping (adaptive and maladaptive) and distress. The interactive effect of maladaptive coping and sleep quality added 2% to the variance in explaining psychological distress (**Table 1**, **Figures 1**, **2**). Moreover, the second part of the hypothesis focused on the interaction of adaptive coping with sleep quality and its impact on psychological distress. The results showed sleep quality to be a significant moderating variable between adaptive coping and distress. This interactive effect of adaptive coping and sleep quality produced a 1% change in psychological distress (**Table 1**, **Figure 2**). These results indicate sleep quality to be a significant moderator in coping and distress and this is a novel finding of the present study. However, empirical evidence has suggested an indirect association between coping and sleep quality (26), and between sleep quality and psychological distress (25, 36). The reasons for these directional findings may be due to the challenging nature of medical studies which demands high attention, commitment, and intellect. Therefore, students feel stressed while pursuing the degree. These stressors become more problematic when other behavioral issues (e.g., disruptive sleep patterns and poor sleep habits) and maladaptive coping get associated with these.

### Limitations

The present study has a number of limitations. The use of self-report measures is limited and subject to various methods biases. In sleep research, other ways of measuring constructs may give a more detailed understanding of problems, including both objective physiological methods and more subjective ones (e.g., sleep diaries). The present study focused on medical college students only and from just three medical colleges with a modest sample size. These students may not be representative of Pakistan’s (or other country’s) medical students. The study also relied on convenience sampling. Future research should involve other (non-medical) students with other age groups to improve generalizability. Future research should focus on other samples such as those in organizational settings.

### Practical implications

Clinical practitioners and educational counsellors can take insight from these findings to promote adaptive coping strategies for professional/vocational trainee students like medical students for overcoming stressors. Moreover, provision of regular psycho-social support or refresher workshops for overcoming stressors and to adapt practically productive life style must be part of their professional or educational training as these will help to improve the mental health of medical students.

## Conclusions

The present study confirmed that both adaptive and maladaptive coping were significantly related to psychological distress and that both adaptive and maladaptive coping had a significant relationship with sleep quality. Moreover, poor sleep quality was also found to be significantly positively associated with psychological distress. Moderation analysis showed that sleep quality was a significant moderator between coping (both adaptive and maladaptive) and psychological distress among medical students.

## Data Availability

The raw data supporting the conclusions of this article will be made available by the authors, without undue reservation.
