# Supplementary material for: Moderating effect of sleep quality in the relationship between coping and distress among medical students
Source: Front Psychiatry. 2024 Oct 2;15:1259842. doi: 10.3389/fpsyt.2024.1259842 (PMC11479903; doi:10.3389/fpsyt.2024.1259842)
Supplement: Supplementary file 1 [file Table1.docx]

Supplementary Table 1*. Correlations between the study variables (N=369)*

| Scales | M | SD | SQ | Dis | CS | AC | MC |
| --- | --- | --- | --- | --- | --- | --- | --- |
| Sleep quality | 23.25 | 6.03 | - |  |  |  |  |
| Distress | 25 | 9.53 | .26** | - |  |  |  |
| Coping | 61.05 | 12.94 | .11** | -.39** | - |  |  |
| Adaptive coping | 37 | 8.2 | -.06* | -.24** | .89** | - |  |
| Maladaptive coping | 24.04 | 6.66 | .14** | .46** | .84** | .51** | - |

*Note.* SQ=sleep quality; CS=coping scale; Dis=distress; MC=maladaptive coping; AC=adaptive coping

** *p*<.01, **p*<.05.
